# Tooth Loss During Supportive Periodontal Care (SPC): A Three Decades Retrospective Study in a University Setting

**DOI:** 10.1111/jcpe.70173

**Published:** 2026-07-19

**Authors:** M. Cyris, P. Bartels, M. Kahl, C. Springer, S. Sälzer, K. Fawzy El‐Sayed, C. E. Dörfer, Li‐Li Zhou, C. Graetz

**Affiliations:** ^1^ Clinic of Conservative Dentistry and Periodontology University Hospital Schleswig‐Holstein Kiel Germany; ^2^ Oral Medicine and Periodontology Department, Faculty of Dentistry Cairo University Giza Egypt; ^3^ Department of Periodontology, The Second Affiliated Hospital, School of Medicine Zhejiang University Hangzhou China

**Keywords:** periodontitis, supportive periodontal care, tooth loss

## Abstract

**Aim:**

This retrospective cohort study evaluate tooth loss and predictors during exceptionally long term supportive periodontal care (SPC), focusing on residual disease severity at SPC initiation.

**Materials and Methods:**

Seventy patients maintained under SPC for > 30 years were analysed using negative binomial regression (log[SPC‐duration] offset) and Cox proportional hazards models (clustered by patient).

**Results:**

Over a mean SPC duration of 36.1 ± 3.0 [30–43] years, 63 patients lost 322 teeth (4.6 ± 4.2 per patient; 0.13 ± 0.12 per patient‐year). Despite advanced baseline disease (stage III/IV: 97%), 35‐year molar survival was 77% for FI 0 versus 51% for FI II–III. Tooth loss rate was significantly associated with PPD ≥ 6 mm (IRR 1.012; 95% CI 1.003–1.021; *p* = 0.009) and molar FI II–III (IRR 1.178; 95% CI 1.050–1.321; *p* = 0.005). A tooth‐level Cox model confirmed elevated hazard for PPD ≥ 6 mm (HR 4.02; 95% CI 2.10–7.69) and FI II–III (HR 2.87; 95% CI 1.61–5.11). However, predictive accuracy was modest (PPV 36%–55%), indicating these markers reflect population‐level risk rather than individual prognosis.

**Conclusion:**

Tooth loss remained low over > 30 years of SPC even for molars with advanced FI, while residual disease severity modulated this risk. Given strong survivorship bias (3.3% of eligible patients), generalisability beyond university‐based specialist care is limited.

## 1 Introduction

Periodontitis is one of the most prevalent chronic inflammatory diseases worldwide and affects a substantial proportion of the adult population (Dye 2012). If left untreated, progressive periodontal destruction leads to attachment and bone loss, functional impairment and, ultimately, tooth loss (Caton et al. 2018; Kocher et al. 2000). Contemporary periodontal therapy consists of active periodontal treatment (APT) followed by individualised supportive periodontal care (SPC)—previously referred to as supportive periodontal therapy (SPT)—as defined in the current EFP S3 clinical practice guideline (Sanz et al. 2020). SPC effectively controls disease progression and substantially reduces tooth loss, allowing retention of the majority of teeth in many patients (Axelsson et al. 2004; Manresa et al. 2018). Long‐term studies consistently highlight regular SPC as a key determinant of periodontal stability and tooth retention (Costa et al. 2014; Graetz et al. 2017). Most longitudinal studies report follow‐up periods of 5–15 years (Costa et al. 2014), whereas data extending beyond two or three decades remain scarce (Agudio et al. 2023). Recent long‐term evidence suggests that outcomes from short‐term studies cannot be directly extrapolated to lifetime periodontal treatment (Fardal et al. 2025). Consequently, evidence on determinants of tooth loss during very long‐term SPC under real‐world clinical conditions remains limited.

Residual periodontal disease severity at the start of SPC may influence long‐term outcomes. In particular, deep residual probing pocket depths (PPDs) and advanced molar furcation involvement (FI) can increase the risk of tooth loss (Matuliene et al. 2008; McFall 1982). The 2018 Classification of Periodontal Diseases emphasised disease severity, complexity and extent, incorporating parameters such as PPD, bone loss and FI into a multidimensional staging framework (Papapanou et al. 2018; Tonetti et al. 2018). Evidence directly linking baseline disease severity to tooth loss during very long‐term SPC remains limited, however. Historical periodontal cohorts with continuous follow‐ups over several decades therefore provide a unique opportunity to investigate these associations under real‐life clinical conditions (Graetz, Schwalbach, et al. 2020). The primary aim of this study was to assess tooth loss during exceptionally long term SPC. The secondary aim was to identify clinical predictors, particularly residual disease severity at SPC initiation, expressed by the proportion of teeth with PPD ≥ 6 mm and by the number of molars with advanced FI (degree II–III).

## 2 Materials and Methods

### 2.1 Sample

**FIGURE 1:**
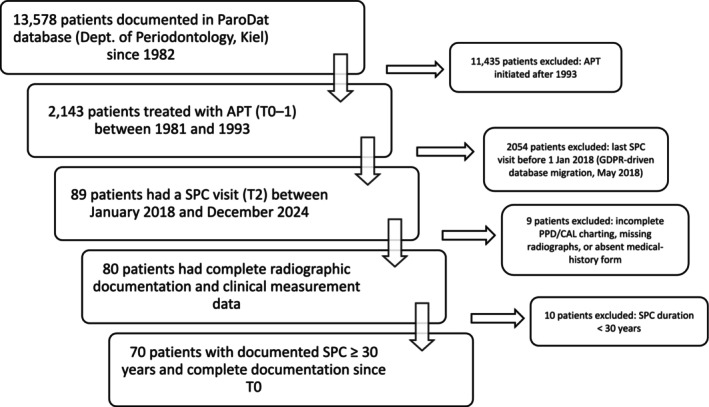
Flowchart of patient recruitment and selection criteria. APT, active periodontal therapy; SPC, supportive periodontal care; T0, baseline (APT start); T1, end of APT; T2, last SPC visit (2018–2024). The 2018 cut‐off reflects the migration of the ParoDat database following the implementation of the General Data Protection Regulation (GDPR) in Germany in May 2018, after which T2 visits were uniformly retrievable.

The present retrospective study included periodontitis patients who had been treated at the Department of Periodontology, Christian‐Albrechts‐University of Kiel, Germany, and who (I) received APT (from the first systematic assessment [T0] to the last active treatment appointment) between 1981 and 1993; (II) had their last documented SPC visit for measuring between the 1 January 2018 and the 31 December 2024; (III) had sufficient parameters documented at T0 to come to a diagnosis according to the 2018 classification; and (IV) received SPC for ≥ 30 years with regular documentation that included intermittently prolonged interruptions appropriate to the extended follow‐up period and complete radiographic documentation enabling assessment of bone loss and treatment course. The reasons for choosing this time period are explained in Section 2.5. No formal sample size calculation was performed, as only a small proportion of patients treated in the 1980s were expected to complete 30+ years of follow‐up. All of them had also been assessed in previous publications (Graetz et al. 2011; Graetz et al. 2017; Graetz, Schwalbach, et al. 2020) and had been followed since then (Figure 1).

### 2.2 Active and Supportive Periodontal Therapy

APT consisted of non‐surgical root debridement and, when indicated, surgical access flap procedures, adjunctive systemic antibiotics (amoxicillin, metronidazole) and, when clinically required, endodontic treatment, splinting and molar root resection. Details of the treatment concept have been described previously (Graetz et al. 2011). SPC followed individualised intervals of 3–12 months and included re‐instruction/re‐motivation of patients, individual oral hygiene as well as professional tooth cleaning with instrumentation of residual pockets and polishing. Details of the treatment concept are described elsewhere (Graetz et al. 2011; Graetz et al. 2017; Graetz, Schwalbach, et al. 2020).

### 2.3 Management of Residual Pockets During SPC

At each recall, sites with PPD ≥ 5 mm and BOP were sub‐gingivally re‐instrumented; non‐responding sites were re‐evaluated for surgical re‐treatment or, for molars with progressive FI, root resection or hemisection. Although this framework remained constant, instrumentation technology evolved over the 30+ year observation period.

### 2.4 Independent Variables

The following variables were available from clinical records, smoking history and dental charting (including third molars); patients were classified per Tonetti et al. (2018), with some modifications as described in detail elsewhere (Graetz et al. 2019):

- Gender and age at T0, T1, and T2.
- Number of missing teeth (at T0, T1 and T2). Note that it was not possible to ascertain the reasons for previous tooth loss (periodontitis or other) in many cases before T0.
- Number of teeth lost during APT (T0–T1) and SPC (T1–T2). In many cases, the reasons for tooth removal were multiple or could not be ascertained (some teeth, for example, were removed *in alio loco* [‘at another location’]).
- Diabetes mellitus (T0, T1, and T2). Other systemic diseases known to influence periodontitis (e.g., coronary heart disease) were not recorded (Genco and Borgnakke 2013).
- Smoking status (T0, T1 and T2) was assessed categorically (never/former [i.e., quit > 5 years ago]/current smoker according Lang and Tonetti 2003).
- PPDs and CAL (T0, T1 and T2) were evaluated at six sites per tooth. CAL was calculated as the sum of PPD plus the distance between the cemento‐enamel junction up to the gingival margin. To classify patients, we only considered the interdental CAL on two non‐adjacent teeth, or, if buccal or oral CAL was ≥ 3 mm, with pocketing > 3 mm (Tonetti et al. 2018).
- Relative radiographic bone loss (BL, in %, T0 and T2) was assessed on peri‐apical radiographic films after digitisation, as described previously (Graetz et al. 2011).
- The highest degree of FI for each molar (T0, T1 and T2) was assessed according to Hamp et al. (1975).
- The extent of periodontitis was classified as localised if ≤ 30% (*n* = 1) or as generalised if > 30% (*n* = 60) of the teeth were affected. No patient with a molar/incisor pattern was available (Tonetti et al. 2018).
- Over the extended observation period, some patients exceeded 12‐month follow‐up in certain years; long‐term SPC adherence was classified as sufficient (≤ 32 months deviation) or insufficient (> 32 months). This threshold corresponds to the cohort‐wide upper quartile of the longest single inter‐recall gap and was chosen a priori to identify patients with at least one substantial interruption (> 2.5 years) without misclassifying the majority as non‐adherent. It represents a pragmatic, dataset‐specific cut‐off. Rather than an externally validated criterion.
- Furthermore, we analysed the data to determine whether patients achieved clinical periodontal stability at T2, defined as the absence of any site with PPD ≥ 5 mm and no site with a PPD of 4 mm exhibiting bleeding on probing (BOP), in accordance with the EFP clinical practice guideline (Sanz et al. 2020) and consistent with the concept of residual disease burden described by Feres et al. (2020).

Further data that may be required for fully applying the new classification, such as the presence of plaque, masticatory dysfunction, bite collapse, drifting or flaring, were not consistently available and were therefore not used (Tonetti et al. 2018). Also, mobility was not considered because of splints in some cases.

### 2.5 Data Management and Statistical Analysis

Data were managed using a dedicated periodontal database (ParoDat, Department of Periodontology, Kiel, Germany) established in 1982 on a database platform (FileMaker, Santa Clara, USA). Following the implementation of the EU General Data Protection Regulation (GDPR) in May 2018, the university hospital restructured all local clinical databases; as a result, only patients with a last documented SPC visit on or after 1 January 2018 could be included, as pre‐2018 records were no longer uniformly retrievable without individual patient re‐contact. All patients provided informed consent for the analysis of their longitudinal clinical data. The study protocol was approved by the Ethics Committee of the Christian‐Albrechts‐University of Kiel (AZ: D489/13; D566/17). Statistical analyses were performed using SPSS Statistics (Version 31.0; IBM Corp., Chicago, IL, USA).

Descriptive statistics were evaluated the at patient and tooth level and are presented as mean ± standard deviation (SD) for continuous variables and as absolute and relative frequencies for categorical variables. Tooth‐level analyses were based on the maximum PPD per tooth at each time point. Tooth survival during APT and SPC was assessed descriptively by the treatment phase and tooth type; changes in PPD categories and FI were evaluated descriptively and stratified by baseline status.

Predictors of tooth loss during SPC were analysed using univariable and multivariable over‐dispersed negative binomial (NB) regression models at the patient level, with the log(SPC‐duration) as an offset. The NB model was selected over Poisson regression based on a likelihood‐ratio test (LR χ^2^ = 65.05, *p* < 0.001; dispersion parameter α = 0.49). Independent variables included age, sex, smoking status, SPC adherence and disease severity at T1 (proportion of teeth with PPD ≥ 6 mm; number of molars with advanced FI degree II–III). Separate multivariable models were constructed for each severity parameter, and a combined model was used to assess the shared variance. A complementary tooth‐level Cox proportional hazards model (*n* = 1792 teeth; 217 events) used robust standard errors clustered by patient. A sensitivity analysis imputed extraction dates for 105 teeth at the T1–T2 midpoint. Results are reported as IRR or HR with 95% CI; *p* < 0.05 was considered significant.

## 3 Results

### 3.1 Baseline Characteristics and Treatment Course

Seventy patients were included, and the basic information of this cohort is presented in Table 1. The duration of APT (T0–T1) was 0.8 ± 0.6 years (range: 0.3–2.6). During APT, 74 teeth were extracted (1.1 ± 2.7 per patient), resulting in a mean of 25.6 ± 3.1 teeth per patient at T1. Extractions occurred in 24 patients (34.3%), whereas 65.7% (*n* = 46) did not lose any tooth during APT. The duration of SPC (T1–T2) was 36.1 ± 2.9 years (range: 30–43). During SPC, 63 patients lost a total of 322 teeth (4.6 ± 4.2 teeth per patient), corresponding to 0.13 ± 0.12 teeth per patient per year. Seven patients (10.0%) experienced no tooth loss, whereas 27 (38.6%), 19 (27.1%), 5 (7.1%) and 12 (17.1%) lost 1–3, 4–6, 7–9 and ≥ 10 teeth, respectively. At T2, 10 patients had received a total of 34 implants. During APT, tooth loss mainly affected molars (64.9%, *n* = 48), followed by anterior teeth (25.7%, *n* = 19) and premolars (9.5%, *n* = 7). During SPC, molars remained the most frequently lost teeth (50.0%, *n* = 161), followed by premolars (26.4%, *n* = 85) and anterior teeth (23.6%, *n* = 76). Among teeth present at T1 and subsequently extracted during SPC, the mean survival time was 21.8 ± 9.6 years (molars 22.6 ± 8.4 years; premolars 21.6 ± 10.2 years; anterior teeth 20.3 ± 11.2 years).

**TABLE 1:** Demographic and clinical characteristics at T0 (admission), T1 (end of APT) and T2 (last SPC visit), with between‐timepoint tests.

Variable	T0	T1	T2	*p* (T0 vs. T1 vs. T2)
Part A: Demographic and treatment variables				
Patients (*n*)	70	70	70	—
Sex, male/female (*n*)	36/34^a^	—	—	—
Age (mean ± SD), years	41.4 ± 7.9	42.0 ± 7.9^b^	77.9 ± 7.9	< 0.001^c^
Active smokers (*n*)	31	30	2	< 0.001^d^
Former smokers (*n*)	39	40	68	—
Never smokers (*n*)	0	0	0	—
Diabetes (any type, *n*)	1	1	7	0.002^d^
Periodontitis stage I/II/III/IV (*n*)	0/2/58/10	—	—	—
Periodontitis grade A/B/C (*n*)	0/18/52	—	—	—
Adherence sufficient (≤ 32 months gaps, *n*/*n*)	—	—	35/35	—
APT duration T0–T1, years	0.6 ± 0.5	—	—	—
SPC duration T1–T2, years	—	—	36.1 ± 2.9	—
Total number of teeth lost APT/SPC	—	74	322	—
Number of teeth lost (%) APT—anterior teeth/premolars/molars (%)	—	19 (25.7)/7 (9.5)/48 (64.9)	—	—
Number of teeth lost (%) SPC—anterior teeth/premolars/molars (%)	—	—	76 (23.6)/85 (26.4)/161 (50.0)	—
Mean survival time extracted teeth, years	—	—	21.8 ± 9.6	—
Part B: Patient‐level periodontal status				
Number of teeth (mean ± SD)	26.7 ± 2.8	25.6 ± 3.4	21.0 ± 6.0	< 0.001^c^
Tooth loss/patient since prev. timepoint (mean ± SD)	—	1.1 ± 2.4	4.6 ± 4.2	—
Mean PPD, mm	5.19 ± 1.38	3.82 ± 1.13	3.36 ± 0.43	< 0.001^c^
Patients with ≥ 1 tooth PPD 1–3 mm, *n* (%)	70 (100.0)	67 (95.7)	70 (100.0)	—
Patients with ≥ 1 tooth PPD 4–5 mm, *n* (%)	70 (100.0)	68 (97.1)	66 (94.3)	—
Patients with ≥ 1 tooth PPD ≥ 6 mm, *n* (%)	70 (100.0)	49 (70.0)	33 (47.1)	< 0.001^d^
Mean BOP, %	42.1 ± 18.6	22.4 ± 15.9	13.8 ± 10.7	—
Mean CAL, mm	6.4 ± 2.6	5.0 ± 2.4	3.6 ± 1.3	< 0.001^c^
Mean BL, % of root length	30.3 ± 15.7	—	31.6 ± 12.9	—
Patients with implants (*n*)	0	0	10	—
Number of implants	0	0	34	—
Part C: Tooth‐level and molar‐level periodontal status				
Total teeth (*n*)	1866	1792	1470	< 0.001^e^
Teeth PPD 1–3 mm, *n* (%)	421 (22.6)	981 (54.7)	1036 (70.5)	0.385^e^
Teeth PPD 4–5 mm, *n* (%)	705 (37.8)	541 (30.2)	380 (25.9)	0.002^e^
Teeth PPD ≥ 6 mm, *n* (%)	740 (39.7)	270 (15.1)	54 (3.7)	< 0.001^e^
Teeth/patient PPD 1–3 mm (mean ± SD)	6.0 ± 5.8	14.0 ± 7.7	14.8 ± 5.8	0.385^e^
Teeth/patient PPD 4–5 mm (mean ± SD)	10.1 ± 5.6	7.7 ± 4.4	5.4 ± 3.5	0.002^e^
Teeth/patient PPD ≥ 6 mm (mean ± SD)	10.6 ± 8.6	3.9 ± 5.3	0.8 ± 1.1	< 0.001^e^
Total molars (*n*)	555	507	346	< 0.001^e^
Mean PPD of molars, mm	5.56 ± 1.77	4.04 ± 1.47	4.02 ± 1.15	0.007^e^
Mean CAL of molars, mm	7.2 ± 2.4	5.9 ± 2.3	4.6 ± 1.5	< 0.001^e^
Mean BL of molars, % root length	34.8 ± 16.9	—	38.6 ± 14.8	< 0.001^f^
Molars FI 0, *n* (%)	321 (57.8)	314 (61.9)	301 (87.0)	—
Molars FI I, *n* (%)	128 (23.1)	113 (22.3)	28 (8.1)	< 0.001^e^
Molars FI II, *n* (%)	65 (11.7)	47 (9.3)	8 (2.3)	< 0.001^e^
Molars FI III, *n* (%)	41 (7.4)	33 (6.5)	9 (2.6)	0.011^e^

Abbreviations: APT, active periodontal therapy; BL, radiographic bone loss; BOP, bleeding on probing; CAL, clinical attachment level; FI, furcation involvement; PPD, pocket probing depth; SPC, supportive periodontal care.

^a^ Sex fixed at baseline.

^b^ Age at T1 approximated as T0 age + APT duration.

^c^ Friedman test (repeated measures, continuous variables across T0/T1/T2).

^d^ Cochran's *Q* test (binary variables).

^e^ Wilcoxon signed‐rank test, T1 vs. T2 paired comparison (T0 tooth‐level data per patient not retrievable from the dataset; T0 vs. T1 comparison therefore not included for Part C variables).

^f^ Wilcoxon signed‐rank test, T0 vs. T2 paired comparison (bone loss not assessed at T1). For Part C rows showing—: either test not significant (molars FI 0: *p* = 0.530; teeth PPD 1–3 mm/teeth/patient PPD 1–3 mm: *p* = 0.385) or comparison not applicable (T1 column for mean BL of molars).

### 3.2 Pocket Probing Depth (PPD) and Bleeding on Probing (BOP)

At baseline, patients had a mean of 10.6 ± 8.6 teeth with PPD ≥ 6 mm. Following APT, this number steadily decreased until T2. Accordingly, the proportion of teeth with shallow PPD (1–3 mm) increased, while the proportion of deep pockets (PPD ≥ 6 mm) decreased (Table 1). When stratified by probing depth at T1, teeth with PPD of 1–3 mm remained largely stable, although 9.6% were extracted by T2. In contrast, teeth with PPD ≥ 6 mm frequently improved among retained teeth, but more than one‐third were ultimately lost during SPC (Table 2). At the patient level, the proportion of patients presenting with at least one PPD ≥ 6 mm decreased (Table 1). Despite an overall reduction in deep pockets, some patients exhibited persistent or increasing numbers of deep sites during SPC (Table 2).

**TABLE 2:** Tooth and molar survival during SPC (T1–T2) stratified by PPD category and furcation involvement at T1 (see Figure 2).

Baseline status at T1	Teeth at T1 (*n*)	Lost during SPC, *n* (%)	Survived to T2, *n* (%)
By PPD category (all teeth)			
All teeth—PPD 1–3 mm	981	94 (9.6)	887 (90.4)
All teeth—PPD 4–5 mm	541	131 (24.2)	410 (75.8)
All teeth—PPD ≥ 6 mm	270	97 (35.9)	173 (64.1)
All teeth—total	1792	322 (18.0)	1470 (82.0)
By furcation involvement (molars only)			
Molars—FI 0	314	82 (26.1)	232 (73.9)
Molars—FI I	113	35 (31.0)	78 (69.0)
Molars—FI II	47	23 (48.9)	24 (51.1)
Molars—FI III	33	21 (63.6)	12 (36.4)
Molars—total	507	161 (31.8)	346 (68.2)

*Note:* Log‐rank: PPD strata χ^2^(2) = 112.4, *p* < 0.001; FI strata χ^2^(2) = 24.3, *p* < 0.001.

Abbreviations: FI, furcation involvement; PPD, pocket probing depth; SPC, supportive periodontal care; T1, end of APT; T2, last SPC visit.

At baseline, all patients presented with BOP at least at one site, and the mean BOP score decreased to T2 (Table 1). BOP remained detectable in 98.6% of patients at T1 and 85.7% at T2. At T2, 46 patients (65.7%) had BOP < 10%. Twelve patients (17.1%) fulfilled the stability criterion according to Sanz et al. (2020), whereas 39 patients (55.7%) showed at least one site with PPD ≥ 5 mm without PPD of 4 mm with BOP. Only two patients (2.9%) presented with a PPD of 4 mm with BOP but without deeper pockets, while both conditions were present in 17 patients (24.3%).

### 3.3 Furcation Involvement (FI) and Molar Survival

At baseline (T0), 555 molars were present, of which 42.2% exhibited FI ≥ I (Table 1). During APT (T0–T1), molar survival remained high but declined with increasing furcation severity. Survival rates were 97.8% for FI 0 molars, 88.3% for FI I, 72.3% for FI II and 80.5% for FI III molars (Table S1). During SPC (T1–T2), FI strongly influenced tooth retention; survival ranged from 73.9% (FI 0) and 69.0% (FI I) to 51.1% (FI II) and 36.4% (FI III) (Table 2). Over the entire observation period (T0–T2), molar survival remained closely associated with baseline furcation status, with markedly lower survival for FI II/III. Among molars surviving to T2, furcation status frequently improved. When stratified by baseline status (T0), improvement rates ranged from 83.3% to 88.0% for FI I–III molars (Table S2). In contrast, molars without FI remained largely stable, although 9.3% deteriorated by T2. A similar pattern was observed during SPC (T1–T2), where improvement rates ranged between 75.0% and 87.2% for FI I–III molars. At T2, the proportion of molars with advanced FI (II–III) was below 5%.

### 3.4 Risk of Tooth Loss During Long‐Term SPC

**FIGURE 2:**
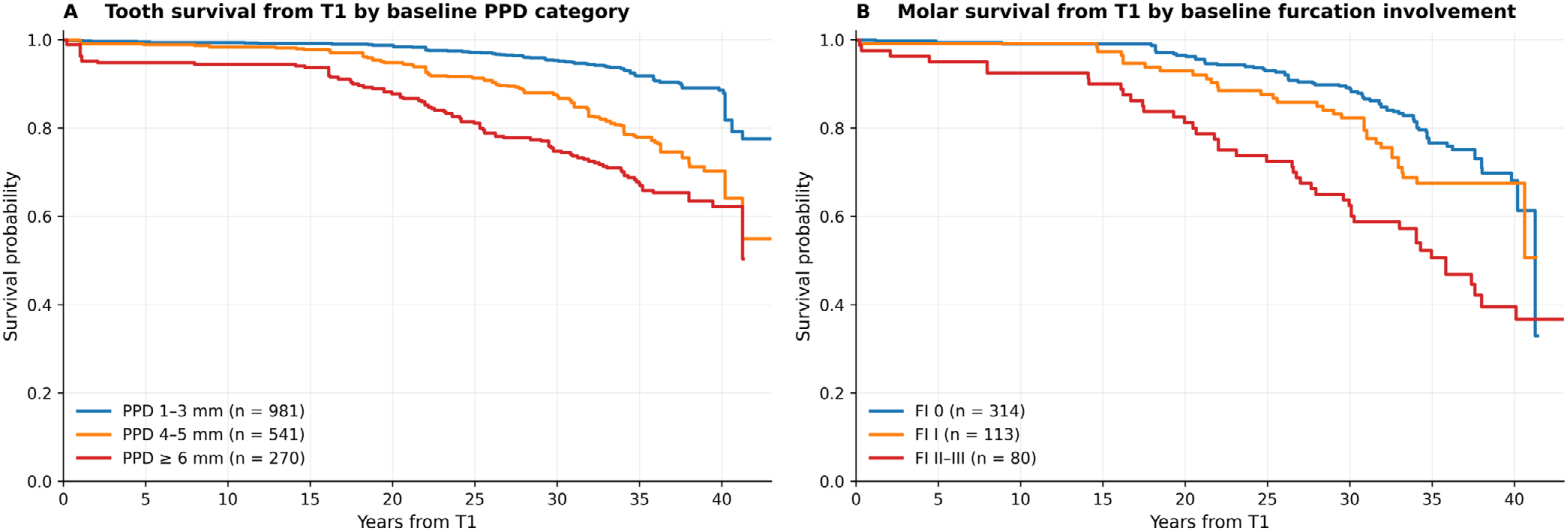
Kaplan–Meier survival curves for teeth present at the end of active periodontal therapy (T1), stratified by baseline disease severity. (A) Tooth survival (all teeth, *n* = 1792) according to pocket probing depth (PPD) category at T1: PPD 1–3 mm (blue, *n* = 981), PPD 4–5 mm (orange, *n* = 541) and PPD ≥ 6 mm (red, *n* = 270). (B) Molar survival (*n* = 507) according to baseline furcation involvement (FI) at T1: FI 0 (blue, *n* = 314), FI I (orange, *n* = 113) and FI II–III (red, *n* = 80). Log‐rank tests: (A) χ^2^(2) = 112.4, *p* < 0.001; (B) χ^2^(2) = 24.3, *p* < 0.001. Time axis reflects years from T1 to tooth extraction or censoring at T2 (last documented supportive periodontal care (SPC) visit, 2018–2024). Primary analysis includes 217 of 322 extracted teeth with an exact extraction date as events; the remaining 105 teeth were right‐censored at T2 (see Table S3 for sensitivity analysis). FI classification according to Hamp et al. (1975). T1, last appointment of active periodontal therapy; T2, last documented SPC visit; PPD, pocket probing depth; FI, furcation involvement; SPC, supportive periodontal care.

In univariable negative binomial regression analyses (with log[SPC‐duration] offset; full results in Table S3), baseline periodontal disease severity at T1 was significantly associated with tooth loss rate during SPC. A higher proportion of teeth with PPD ≥ 6 mm increased the tooth loss rate (IRR 1.010 per percentage point; 95% CI 1.001–1.019; *p* = 0.027), as did a higher number of molars with advanced FI (IRR 1.150 per molar; 95% CI 1.030–1.284; *p* = 0.013). In multivariable analyses adjusted for age, smoking status and adherence, both severity parameters remained independently associated with tooth loss rate. The proportion of teeth with PPD ≥ 6 mm at T1 (IRR 1.012; 95% CI 1.003–1.021; *p* = 0.009) and each additional molar with advanced FI (IRR 1.178; 95% CI 1.050–1.321; *p* = 0.005) increased the annual tooth loss rate. In a combined model including both variables, neither reached significance (PPD: IRR 1.007, *p* = 0.131; FI: IRR 1.133, *p* = 0.051), indicating shared variance between these markers of residual disease severity (Table 3). The tooth‐level Cox model confirmed substantially elevated hazard for teeth with PPD 4–5 mm (HR 2.24; 95% CI 1.39–3.60; *p* < 0.001) and PPD ≥ 6 mm (HR 4.02; 95% CI 2.10–7.69; *p* < 0.001) at T1 relative to PPD 1–3 mm (Figure 2). Kaplan‐Meier estimates indicated 35‐year molar survival of approximately 77% (FI 0), 67% (FI I) and 51% (FI II–III; Figure 2B). Molars with FI II–III showed HR 2.87 (95% CI 1.61–5.11; *p* < 0.001) relative to FI 0, while FI I was not significant (HR 1.38, *p* = 0.328). The proportional‐hazards assumption was met (global χ^2^ = 3.06, df = 6, *p* = 0.80). Sensitivity analyses with imputed extraction dates preserved direction and significance of all effects (Table 4 and Table S4). Predictive accuracy at the individual‐tooth level was modest: PPV of PPD ≥ 6 mm for subsequent extraction was 35.9% (sensitivity 30.1%, specificity 88.2%), and PPV of FI II–III for molar extraction was 55.0% (sensitivity 27.3%, specificity 89.6%), confirming that these markers identify elevated population‐level risk but are not deterministic at the individual level (Table 5).

**TABLE 3:** Multivariable negative binomial regression for tooth loss during SPC (T1–T2), patient level.

Predictor (T1)	Model A %PPD ≥ 6 mm IRR	95% CI	*p*	Model B FI II–III IRR	95% CI	*p*	Combined IRR	95% CI	*p*
Age (per year)	1.025	0.999–1.053	0.064	1.023	0.996–1.050	0.097	1.026	0.999–1.053	0.054
Active smoker (vs. former smoker)	1.025	0.677–1.552	0.907	0.955	0.633–1.440	0.825	0.947	0.631–1.422	0.793
Insufficient SPC adherence (> 32 months)	0.900	0.596–1.358	0.614	1.144	0.750–1.745	0.532	1.075	0.703–1.645	0.739
% teeth PPD ≥ 6 mm at T1 (per %‐pt)	1.012	1.003–1.021	0.009	—	—	—	1.007	0.998–1.017	0.131
Molars FI II–III at T1 (per molar)	—	—	—	1.178	1.050–1.321	0.005	1.133	1.000–1.284	0.051

*Note:* Log (SPC‐duration) offset; IRR per patient‐year. Overdispersion: Variance/mean = 3.85; LR χ^2^ = 65.05, *p* < 0.001; α = 0.49. Univariable results are in Table S3. Age, sex, smoking status and SPC adherence were included as covariates in all multivariable models.

Abbreviations: CI, 95% confidence interval; FI, furcation involvement; IRR, incidence rate ratio per patient‐year; PPD, pocket probing depth; SPC, supportive periodontal care.

**TABLE 4:** Cox proportional‐hazards model for tooth survival from T1 (*n* = 1792 teeth, 70 patients; 217 events).

Predictor	HR	95% CI	*p*
PPD 4–5 mm at T1 (vs. 1–3 mm)	2.24	1.39–3.60	< 0.001
PPD ≥ 6 mm at T1 (vs. 1–3 mm)	4.02	2.10–7.69	< 0.001
FI I at T1 (vs. FI 0)	1.38	0.72–2.64	0.328
FI II–III at T1 (vs. FI 0)	2.87	1.61–5.11	< 0.001
Molar (vs. anterior)	1.74	0.88–3.42	0.110
Premolar (vs. anterior)	1.62	1.00–2.62	0.048

*Note:* Cluster‐robust SE by patient. Reference: PPD 1–3 mm; FI 0; anterior tooth. Proportional hazards assumption met: global χ^2^ = 3.06, df = 6, *p* = 0.80. Sensitivity analysis (105 imputed teeth): see Table S4.

Abbreviations: CI, confidence interval; FI, furcation involvement; HR, hazards ratio; PPD, pocket probing depth; SE, standard error.

**TABLE 5:** Predictive accuracy of baseline disease‐severity markers for tooth loss during SPC (T1–T2).

Baseline marker (T1)	*n* flagged	Sensitivity	Specificity	PPV	NPV
PPD ≥ 6 mm at T1 (all teeth)	270/1792	30.1% (97/322)	88.2% (1297/1470)	35.9% (97/270)	85.2% (1297/1522)
FI II–III at T1 (molars only)	80/507	27.3% (44/161)	89.6% (310/346)	55.0% (44/80)	72.6% (310/427)

*Note:* Although both markers are significant risk factors at population level (Table 3), individual‐tooth PPV is modest (35.9%–55.0%), confirming they are prognostic markers rather than deterministic predictors.

Abbreviations: FI, furcation involvement; NPV, negative predictive value; PPD, pocket probing depth; PPV, positive predictive value; SPC, supportive periodontal care.

## 4 Discussion

In this university‐based cohort, we found that long‐term SPC enables sustained periodontal stability and tooth retention even in initially severe periodontitis, extending prior evidence to observation periods exceeding three decades (Agudio et al. 2023; Graetz, Baumer, et al. 2020). Our annual tooth loss rate (0.13 teeth/patient/year) falls within the range reported in multi‐centre German university data (0.11–0.23; Graetz, Baumer, et al. 2020; Pretzl et al. 2018).

### 4.1 Temporal Distribution of Tooth Loss: Comparison With Existing Evidence

A key finding of the present study is the late accumulation of tooth loss during SPC, with relatively few extractions in the early years of maintenance and increasing losses after extended follow‐up. Similar temporal patterns have already been described in long‐term cohort studies, in which tooth loss occurred predominantly in later phases of dental care or at older ages, even under long‐term preventive programmes (Axelsson et al. 2004; Graetz et al. 2011; Konig et al. 2002).

### 4.2 Clustered Tooth Loss Events in the Context of Previous Studies

These temporal patterns complement prior reports focusing on cumulative tooth loss rates by characterizing the timing of extraction events within the maintenance phase (Carvalho et al. 2021). While earlier studies reported increased tooth loss in older age groups or after prolonged maintenance (Konig et al. 2002; Loe et al. 1986), the present study suggests that this acceleration may occur as clustered extraction events rather than as a gradual increase. This pattern resembles observations from long‐term cohorts of aggressive periodontitis, where tooth loss accelerated in later phases despite maintenance (Baumer et al. 2020). Beyond cumulative tooth loss, supplementary analyses revealed clustered tooth loss events predominantly after > 20 years of maintenance (Table S5), consistent with temporal patterns reported by others (Axelsson et al. 2004; Fardal et al. 2025; Graetz et al. 2011). Annual tooth loss rates in our cohort (0.015/0.136/0.342 for low/moderate/high groups) were broadly comparable to those reported by Fardal et al. (2025) with 0.03/0.16/0.38; however, in contrast, the temporal shift in our cohort appeared more gradual. This pattern likely reflects cumulative biological susceptibility and ageing‐related factors rather than insufficient maintenance (Graetz et al. 2013).

### 4.3 Clinical Interpretation in Light of the Existing Literature

Taken together, these findings confirm the effectiveness of long‐term SPC while demonstrating that late clustered tooth loss represents natural limits of preservation in ageing periodontal patients rather than failure of therapy (Konig et al. 2002). Persistent PPD ≥ 6 mm and FI have previously been identified as predictors of tooth loss during SPC (Rieger et al. 2024), consistent with earlier observations by Salvi et al. (2014) and Ramseier et al. (2019), as well as with the present findings. In addition to the temporal distribution of tooth loss, baseline periodontal disease severity at SPC initiation remained an important determinant of long‐term outcomes. However, tooth‐level analyses demonstrated considerable stability and improvement even among high‐risk teeth with PPD ≥ 6 mm, with 60% improving and only 36% being extracted. These findings suggest that such parameters should be interpreted as markers of increased long‐term risk rather than deterministic indicators of inevitable failure (Helal et al. 2019). This interpretation is further supported by machine learning models showing that established periodontal risk factors achieve only moderate prognostic accuracy after external validation (AUC 0.62–0.82) (Krois et al. 2019).

The prospective study by Hasan et al. (2024) on tooth loss during SPC provides a useful contemporary benchmark. Consistent with our findings, their shorter term to mid‐term prospective cohort identified residual disease severity as a key determinant of tooth loss during SPC, with deep residual pockets and advanced FI emerging as significant risk markers. Convergent results across both study designs strengthen the clinical relevance of these predictors. Divergent aspects include differences in cohort composition (university specialist care vs. mixed practice settings), follow‐up duration (their study covering a shorter observation period compared with our 30+ years) and the fact that their prospective design allowed for more standardized documentation of extraction reasons—a limitation explicitly acknowledged in the present study. Together, these findings suggest that although baseline predictors and residual disease severity provide robust prognostic guidance at the population level across different SPC settings and observation periods, they are insufficiently accurate for direct clinical decision making on individual teeth and should therefore be integrated with tooth‐specific clinical and radiographic re‐evaluation during SPC.

### 4.4 Limitations

Several limitations apply. The retrospective design limits causal inference; tooth loss reflects multifactorial extraction decisions (including prosthetic considerations and teeth removed in *alio loco*), precluding attribution to periodontitis alone (Graetz et al. 2013). Other relevant factors (occlusal loading, parafunctional habits, systemic changes) could not be fully evaluated. The absence of never‐smokers restricts smoking comparisons to active versus former smokers. Most importantly, only 70 of 2143 eligible patients (3.3%) completed 30+ years of follow‐up: survivorship bias limits generalisability beyond university‐based specialist care.

## 5 Conclusion

In this highly selected university cohort, SPC preserved most teeth for more than 30 years, even in patients with initially severe periodontitis. Residual periodontal disease severity at the start of SPC, particularly deep residual PPD and advanced FI, modulated this risk. These baseline predictors identified at the population level provide useful prognostic guidance but are not sufficiently accurate for direct clinical decisions on individual teeth and should be combined with tooth‐specific clinical and radiographic re‐evaluation during SPC. Given the strong survivorship bias inherent in this cohort, the favourable outcomes cannot be directly extrapolated to the broader periodontitis population. These findings support prioritizing thorough residual disease control before SPC entry and individualizing risk‐adapted maintenance strategies according to residual PPD and furcation status.

## Funding

The authors have nothing to report.

## Conflicts of Interest

The authors declare no conflicts of interest.

## Supporting information


**Table S1:** Molar survival during active periodontal therapy (APT, T0–T1) stratified by baseline furcation involvement (FI) at T0.
**Table S2:** Furcation involvement (FI) change patterns for molars surviving to T2: T0→T2 (entire observation period) and T1→T2 (SPC period only).
**Table S3:** Univariable negative binomial regression analyses for tooth loss during SPC (T1–T2), patient level. Log (SPC‐duration) offset included.
**Table S4:** Sensitivity analysis for the Cox proportional hazards model: primary analysis (217 teeth with known extraction date as events; 105 right‐censored) versus sensitivity analysis (322 events; 105 teeth imputed at midpoint T1–T2).
**Table S5:** Temporal distribution of documented tooth loss during SPC (T1–T2) among patients with known exact extraction date (*n* = 217 of 322 extracted teeth).

## Data Availability

The data that support the findings of this study are available on request from the corresponding author. The data are not publicly available due to privacy or ethical restrictions.
