# Supplementary material for: Tooth Loss During Supportive Periodontal Care (SPC): A Three Decades Retrospective Study in a University Setting
Source: J Clin Periodontol. 2026 Jul 19;53(9):1481–90. doi: 10.1111/jcpe.70173 (PMC13477840; doi:10.1111/jcpe.70173)
Supplement: Supplementary file 1 [file JCPE-53-1481-s001.docx]

**Online Supplemental Material**

*Tooth loss during supportive periodontal care (SPC) – JCP MS-ID 7359818*

Contents: Supplemental Tables S1–S5

**Tooth-level survival analysis during supportive periodontal care (SPC)**

During SPC, tooth loss was recorded at the tooth level. As reported in Table 1 of the main manuscript, a total of 322 teeth were extracted during SPC. For 217 of these teeth, an exact date of extraction was available, allowing for longitudinal time-to-event analyses. For the primary survival analysis, only teeth with a documented exact extraction date were included as events (n = 217); the remaining 105 teeth without an exact date were right-censored at the end of follow-up (T2). A sensitivity analysis re-introduced these 105 teeth as events imputed at the midpoint between T1 and T2 (Supplemental Table S4). Teeth remaining present until T2 were treated as censored observations. Time to tooth loss was calculated from T1 to the documented extraction date; follow-up time for censored teeth was calculated from T1 to the last documented SPC visit. This analysis demonstrated that tooth loss during SPC was not evenly distributed over time: only a limited number of extractions occurred during the early years, while the majority occurred after more than 20 years of SPC (Supplemental Table S5).

**Supplemental Table S1. Molar survival during active periodontal therapy (APT, T0–T1) stratified by baseline furcation involvement (FI) at T0.**

| **FI at T0** | **Molars at T0 (n)** | **Survived to T1 (n)** | **Lost during APT, n (%)** | **Survival rate (%)** |
| --- | --- | --- | --- | --- |
| FI 0 | 321 | 314 | 7 (2.2) | 97.8 |
| FI I | 128 | 113 | 15 (11.7) | 88.3 |
| FI II | 65 | 47 | 18 (27.7) | 72.3 |
| FI III | 41 | 33 | 8 (19.5) | 80.5 a |
| **Total** | **555** | **507** | **48 (8.6)** | **91.4** |

*a FI III: interpret with caution (small n). FI = furcation involvement; APT = active periodontal therapy; T0 = admission; T1 = end of APT.*

**Supplemental Table S2. Furcation involvement (FI) change patterns for molars surviving to T2: T0→T2 (entire observation period) and T1→T2 (SPC period only).**

| **Baseline FI** | **n molars surviving** | **Worsened** | **Improved to FI I** | **Improved to FI 0** | **Stable** |
| --- | --- | --- | --- | --- | --- |
| ***T0 → T2 (entire observation period)*** | | | | | |
| FI 0 at T0 | 321 | 29 (9.0) ↑ | 0 | 0 | 292 (91.0) |
| FI I at T0 | 113 a | — | 94 (83.2) ↓ | 9 (8.0) ↓↓ | 10 (8.8) |
| FI II at T0 | 47 a | — | 41 (87.2) ↓↓ | — | 6 (12.8) |
| FI III at T0 | 33 a | — | 29 (88.0) ↓↓↓ | — | 4 (12.0) |
| ***T1 → T2 (SPC period only)*** | | | | | |
| FI 0 at T1 | 314 | 27 (8.6) ↑ | 0 | 0 | 287 (91.4) |
| FI I at T1 | 78 a | — | 60 (76.9) ↓ | 6 (7.7) ↓↓ | 12 (15.4) |
| FI II at T1 | 24 a | — | 21 (87.5) ↓↓ | — | 3 (12.5) |
| FI III at T1 | 12 a | — | 9 (75.0) ↓↓↓ | — | 3 (25.0) |

*Only molars surviving to the respective endpoint are included. Arrows: ↑ = worsening; ↓/↓↓/↓↓↓ = improvement by one/two/three FI categories. Stable = no change. a Only molars present at T0 or T1 respectively. FI = furcation involvement; T0 = admission; T1 = end of APT; T2 = last SPC visit; SPC = supportive periodontal care.*

**Supplemental Table S3. Univariable negative binomial regression analyses for tooth loss during SPC (T1–T2), patient level. Log(SPC-duration) offset included.**

| **Predictor** | **IRR** | **95% CI** | **p** |
| --- | --- | --- | --- |
| Age at T0 (per year) | 1.017 | 0.989–1.045 | 0.240 |
| Sex — male (vs female) | 1.019 | 0.663–1.567 | 0.931 |
| Active smoker (vs former) | 1.044 | 0.677–1.609 | 0.846 |
| Insufficient SPC adherence (>32 mo gap) | 0.915 | 0.596–1.406 | 0.686 |
| % teeth with PPD ≥ 6 mm at T1 (per %-point) | 1.010 | 1.001–1.019 | 0.027 |
| Molars with FI II–III at T1 (per molar) | 1.150 | 1.030–1.284 | 0.013 |

*IRR = incidence rate ratio per patient-year; CI = 95% confidence interval. Multivariable counterparts (adjusted for age, smoking, and SPC adherence) are in Table 3 of the main manuscript.*

**Supplemental Table S4. Sensitivity analysis for the Cox proportional-hazards model: primary analysis (217 teeth with known extraction date as events; 105 right-censored) vs. sensitivity analysis (322 events; 105 teeth imputed at midpoint T1–T2).**

| **Predictor** | **Primary HR** | **95% CI** | **p** | **Sensitivity HR** | **95% CI** | **p** |
| --- | --- | --- | --- | --- | --- | --- |
| PPD 4–5 mm (vs 1–3 mm) | 2.24 | 1.39–3.60 | <0.001 | 2.26 | 1.41–3.62 | <0.001 |
| PPD ≥ 6 mm (vs 1–3 mm) | 4.02 | 2.10–7.69 | <0.001 | 3.28 | 1.95–5.52 | <0.001 |
| FI I (vs FI 0) | 1.38 | 0.72–2.64 | 0.328 | 1.08 | 0.65–2.27 | 0.547 |
| FI II–III (vs FI 0) | 2.87 | 1.61–5.11 | <0.001 | 2.00 | 1.22–3.29 | 0.006 |
| Molar (vs anterior) | 1.74 | 0.88–3.42 | 0.110 | 1.85 | 0.97–3.52 | 0.062 |
| Premolar (vs anterior) | 1.62 | 1.00–2.62 | 0.048 | 1.58 | 1.00–2.49 | 0.048 |

*HR = hazard ratio; CI = confidence interval. Direction and significance of all effects preserved across analyses. Largest change: FI II–III HR 2.87 → 2.00 (−30%). Primary analysis results are reported in Table 4 of the main manuscript.*

**Supplemental Table S5. Temporal distribution of documented tooth loss during SPC (T1–T2) among patients with known exact extraction date (n = 217 of 322 extracted teeth).**

| **Time since start of SPC (T1)** | **Teeth lost, n (%)** | **Patients with extractions (n)** | **Patients with >3 extractions in interval (n)** |
| --- | --- | --- | --- |
| 0–5 years | 25 (11.5) | 9 | 2 |
| 5–10 years | 5 (2.3) | 3 | 0 |
| 10–20 years | 43 (19.8) | 18 | 4 |
| 20–30 years | 105 (48.4) | 31 | 14 |
| 30–40 years | 39 (18.0) | 16 | 5 |
| >40 years | 0 (0.0) | 0 | 0 |
| **Total** | **217 ^a^ (100.0)** | **42 ^b^** | **30** |

*^a^ Only teeth with an exact documented extraction date are included (217 of 322 total extractions during SPC; the remaining 105 teeth are right-censored at T2 in the primary analysis). ^b^ Patients who experienced tooth loss in more than one time interval are counted in each relevant interval; the patient column therefore does not sum to the total number of patients with extractions (n = 63).*
